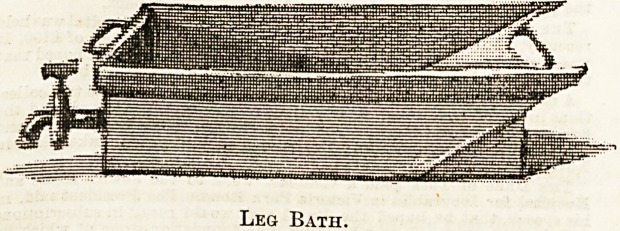# Practical Departments

**Published:** 1894-07-28

**Authors:** 


### PRACTICAL DEPARTMENTS.

### THE HOSPITAL BATH-ROOM.

The bath-room is naturally a very important adjunct to the hospital ward, more especially so, perhaps, because so many of the patients therein are not at all in the habit of looking upon bath-rooms or baths at all in the light of a necessary of life. But sister and nurses take a different view of the matter, as

Hospital Bath-room.

an inspection of the bath-room and its accessories at any well-managed institution will amply testify.

For appearance and the advantage of easy cleansing and keeping in good order, porcelain baths are undoubtedly the best possible, but they are more expensive to provide in the first instance, and there is the risk of damage with any rough usage. Enamelled iron is most generally used, therefore, for institutional purposes. The bath shown in the accompanying sketch is of this latter type. It is advisable to have such a

Leg Bath.

bath as the one illustrated (which is of the simplest and least expensive make, though admirable in every way for its purpose) raised from the ground, so as to allow of no corners for accumulation of dust, &c. It should also, of course, be placed in the centre of the space, so as to permit free access on all sides—a most necessary condition in the bathing of helpless patients. Height is another point which requires a little consideration. The bath should be neither so high as to be awkward for the patient, nor low enough to cause unnecessary stooping and effort on the part of the nurse.

Messrs. Cliff and Sons, of Wortley and London, who have supplied many hospitals and institutions, have every kind of bath in stock, and their work is thorough and good. The bath-room sketched here is one of those fitted by this firm.

Our second drawing shows a special form of bath which will be familiar to nurses as a "leg" or "arm" bath, used for surgical cases where either limb is ordered prolonged immersion. It is a sufficiently difficult matter in the first case to so arrange a bath of this description as to keep a leg covered by the water up to, say, the knee. The shape here given is the best adapted for such purpose, the sloped end facilitating matters as much as possible. The water can be run off by means of the tap at the lower end, without moving the bath itself, and the contents thus changed as often as need be with no difficulty. The edge of these, as equally of the large baths, are best when rounded in shape as much as possible, to avoid any chance of contact with sharp edges.

## Figures and Tables

**Figure f1:**
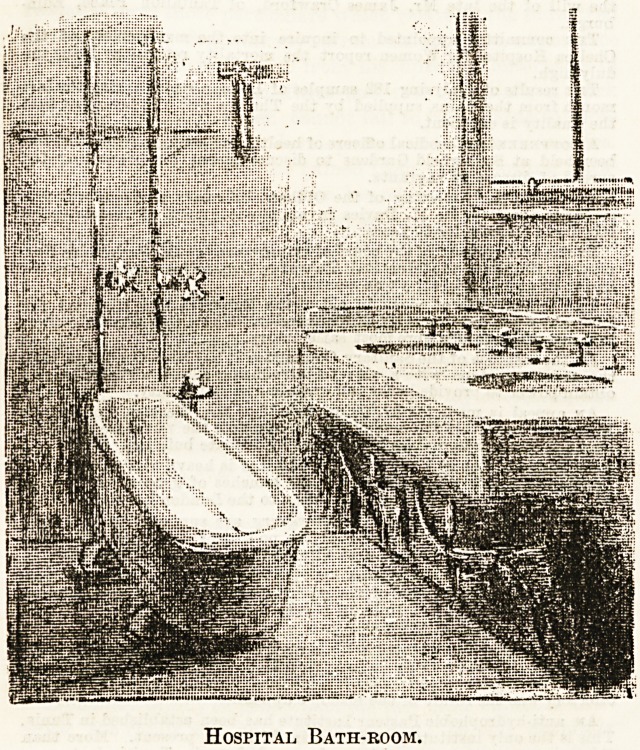


**Figure f2:**